# Analecta Minora, or Minor Periscope

**Published:** 1825-04-01

**Authors:** 


### XIII.

### ANALECTA MINORA,

OR

### *MINOR PERISCOPE.*

——— sparsa Colligens.

1. *Extinction of Fire.* The Parisian Council of Health report, that powdered sulphur, thrown on the fire in the grate, extinguishes, very quickly, a fire in the chimney above. A pound of the substance, sprinkled on the fire, is said to be quite sufficient for this purpose.

2. *Italian Remedies.* It is not in modern times alone that the Italians have surprised their brethren by the large doses of their medicines. Some 90 years ago the physicians of that country took it into their heads that cold water was the sovereign remedy for fevers and other complaints. They therefore gave from 15 to 25 pints of iced water daily to each patient labouring under fever, small-pox, dropsy, &c !—*Commerc. Nov.* 1736. *Hebd.* 8. Tartar emetic is now the catholicon there.

3. *Pressure on the Perineum.* We observed, in one of the numbers of this Journal, that we did not conceive pressure on the perineum in labour to be a preventive of laceration of that part. Our observations seem to be partly confirmed by Dr. Collins, who (Med. and Phys. Journ. February) thus remarks :—" It has long been my opinion that the principal object of a prudent practitioner ought to be, to press the head of the fœtus, in its passage during a pain, against the arch of the pubes, and thus to diminish the pressure on the perineum. This mode of proceeding I consider much more eligible than pressure made on the perineum itself."—*Loco citato.*

4. *Therapeutic Agents.** M. Bally has reported from La Charité on the operation of certain medicinal substances, as observed by him at the above-mentioned institution. These observations we shall condense for the benefit of our readers.

1. *Sulphate of Quinine.* When vomits, leeches, or general blood-letting have not arrested intermittent fevers, our author has had recourse to this powerful tonic—always examining first the state of the mucous membrane of the stomach—this medicine being capable, he thinks, of kindling an inflammation of that part, if given unguardedly. He observes, however, that the sulphate of quinine is much less irritating to the bowels than the bark in substance—and, truly, so it is. In fact, we hardly believe that the sulphate of quinine has any irritating effect on the coats of the stomach, but rather a tonic and soothing influence. Our author cites a case of a child, four years of age, who each day, at a certain hour, experienced such violent colicky pains, that he lay screaming and rolling on the floor for some time. The sulphate of quinine quickly dissipated this affection, without producing any irritation in the stomach or bowels.

* M Bally. Revue Med. Decembre, 1824.

2. M. Bally has administered the *Acetate of Morphine* in rheumatisms (acute) and with much success, by gradually increasing the dose. In one case, however, metastasis to the brain took place, and the cure was protracted to the twentieth day. It was in the month of June that he commenced his trials with *morphine*. He was at first led to think, with the chemists, that the salifiable base, not being soluble without acid, was incapable of exciting any action on the living body. He, therefore, gave it in doses of half a grain night and morning to eight or ten patients ; and was not a little surprised to find that it acted with quite as much energy as the acetates or sulphates of the same substance. He was, in consequence, obliged to proceed with more caution, and to administer the medicine in doses of an eighth of a grain, or even less, every twelve hours. In these doses it produced a tranquil sleep. He relates cases of rheumatism and other painful diseases, where the morphine appeared to have an excellent effect.

3. *Extract of Lettuce.* This has been tried as a substitute for opium, by our author, and is much praised. But the preparation is more used and better known here than on the Continent, and, therefore, we need not stop to notice M. Bally's observations.

4. *Nitrate of Potash.* This medicine has been given in large doses, by M. Bally, in dropsies. It acted as a powerful diuretic. We shall introduce a short case in illustration. A young man, 25 years of age, at the close of an intermittent fever, treated by repeated application of leeches, became affected with dropsy, accompanied by the following symptoms:—dryness, discoloration, and flaccidity of the skin—puffy state of the face—extreme whiteness of the conjunctiva, which appeared entirely deprived of blood-vessels—constant thirst—high-coloured urine, and scanty—depression of spirits—disturbed sleep. The patient was twice purged with cream of tartar, but the symptoms remained the same. He was generally anasarcous, but more so in some places than in others. The circulation was very languid—the pulse slow and feeble—palpitations of the heart—tongue pale and covered with mucus. Nitrate of potash, in doses of two drachms in the day, and continued for a fortnight, produced a most copious diuresis, and the dropsy entirely disappeared.

---

5. *Tinct. Flor. Colchici.** It is a presumptive proof that colchicum autumnale is a valuable medicine, when we find different preparations of it extolled, each as superior to all the others. It is, in truth, a most important medicinal agent in many complaints, but especially in gout, rheumatism, and inflammatory affections of the respiratory organs.

Mr. Bushell, a very intelligent practitioner of this Metropolis, has, for some time past, employed the flowers of colchicum in tincture, and speaks highly of their good effects. The tincture is prepared as follows.

R Flor. Colch. exsiccat et cont. ℥j.
Spir. Tenuioris octarium unum,
macera per dies septem, et cola.

We observe, by the cases brought forward in Mr. Bushell's report, that he gives this tincture in drachm doses every six hours, lessening the quantity as the symptoms abate. In these doses, the medicine keeps up a pretty sharp action on the bowels and skin. We think this tincture must be

---

* Mr. Bushell. Med. Repos. February, 1825.

weaker than the vinum colchici, for the latter could not be given in such quantities, without risk of considerable action on the stomach and bowels. The cases adduced by Mr. Bushell were principally acute rheumatism, and one of chronic iritis. They prove the efficacy of the medicine unequivocally, as it was given in simple distilled water, without any other therapeutic agent, and soon dissipated the complaints for which it was administered.

The flowers of colchicum, which are produced in September, possess the acro-narcotic odour, and contain a considerable quantity of acrid mucilaginous juice, which renders them difficult to dry. Not only the petals, but the whole flower-tubes should be gathered for use.

---

6. *Old Ulcers.* Although Wiseman has recommended laced-stockings in some cases of chronic ulcers on the legs, yet we believe it was only about half a century ago that the practice of pressure and bandaging, *i. e.* Baynton's method, was first brought forward, by Mr. Else, Surgeon to St. Thomas's Hospital. He acknowledges that he learnt the method from an apothecary in Half-Moon Street, Piccadilly, who became celebrated for curing old ulcers of the legs by tight-bandaging, and *five drops* of a secret nostrum twice a day. Mr. Else offered to give the apothecary the contract for supplying the hospital with the secret remedy, but the apothecary wisely observed that the medicine would not cure old ulcers unless administered by himself! Mr. Else then took to the tight bandaging, by means of a roller applied from the foot to the knee, with a thin piece of lead laid over the sore, which was first dressed with cerate. The bandage was laid on so tight as to make the leg feel numb, and the success was general. Every one may see that this is, to all intents and purposes, the method from which Baynton derived so much credit.—*See Medical Observations and Enquiries, for* 1772, *vol* iv. *p.* 352.

---

7. *Volatile Oil of Cubebs.* We are informed by Mr. Battley that, upon a recent examination of cubebs, he found an *extremely volatile oil,* in the proportion of one ounce and a quarter to the pound. In this oil he apprehends the virtue of the drug to reside,—and, if so, the great uncertainty of its effects on disease may be readily accounted for by the supposition that, in many instances, the medicine, when administered, has contained the oil, while, in other instances, the oil may have escaped. If the virtue of the substance should be found to reside in the oil, we apprehend the discovery will be a very important one, as we shall then have a remedy for gonorrhœa in as small a compass as we have for ague in arsenic or quinine. We advise trials to be immediately made with the oil.

---

8. *Intro-Uterine Vociferation.* Some incredible stories have been related of infants crying in the womb. God knows they cry enough after emerging from their prison house! We have applied the term *incredible* to the former instances, because it was said that the infants cried while head, body, and extremities were in the uterus—a thing almost beyond belief. In the present instance, M. Marc has read to the Royal Academy of Medicine, the case of a head-presentation, where it was deemed proper to turn, in consequence of pelvic deformity. When the lower extremities and body were extracted, the child cried distinctly, three times, before the head was delivered. The child died almost immediately after birth. Here there was no compression of the uterus on the body, and, it is *possible,* some air might be in the cavity of the womb after turning, which might allow the act of imperfect respiration, and empower the infant to cry.

